# Parental ADHD as a Mechanistic Barrier to Behavioral Parent Training Implementation: An Intergenerational Framework for Addressing Childhood ADHD

**DOI:** 10.3390/brainsci16050495

**Published:** 2026-04-30

**Authors:** Lauren M. Friedman, Gabrielle Fabrikant-Abzug, Lindsay C. Chromik

**Affiliations:** Department of Psychology, REACH Institute, Arizona State University, Tempe, AZ 85251, USA

**Keywords:** attention-deficit/hyperactivity disorder (ADHD), parental ADHD, behavioral parent training, parenting

## Abstract

**Highlights:**

**What are the main findings?**

- Behavioral parent training (BPT) is an effective treatment for childhood ADHD, but many families see limited or short-lived benefits.
- When parents also have ADHD, they often have difficulty consistently using BPT strategies, which reduces treatment effectiveness.

**What are the implications of the main findings?**

- Improving BPT outcomes for childhood ADHD likely requires an intergenerational approach that directly addresses parental ADHD.
- Integrating targeted supports for parental ADHD that explicitly address barriers to skill implementation (e.g., through CBT-informed strategies, digital tools, and tailored intervention content) may improve skill use, treatment durability, and real-world effectiveness.

**Abstract:**

Behavioral parent training (BPT) is a front-line psychosocial treatment for childhood ADHD, yet its real-world effectiveness is often constrained by parents’ ability to consistently implement learned strategies. Parental ADHD is a prevalent and mechanistically important factor shaping both parenting behavior and child treatment response. Among parents with ADHD, deficits in executive functioning and emotion regulation, abilities essential for consistent and effective BPT implementation, often interfere with parents’ ability to apply learned strategies. Consequently, parental ADHD predicts reduced in-home skill use and attenuated child treatment gains, positioning it as a potentially critical, treatment-relevant risk factor. This narrative review synthesizes evidence on the intergenerational transmission of ADHD-related impairments, the impact of parental ADHD on parenting practices, and the role of parental ADHD as a moderator of BPT outcomes. We also examine existing approaches to addressing parental ADHD within the context of child BPT, including both pharmacological and psychosocial strategies, and evaluate their implications for parenting and child response. Building on this, we propose an intergenerational reconceptualization of psychosocial care for childhood ADHD in which parental functioning is routinely assessed and supported within BPT. Promising directions include integrating CBT-informed strategies to scaffold parents’ cognitive and regulatory processes, incorporating digital health tools that provide just-in-time guidance at the point of parenting performance, and tailoring BPT emphasis for families affected by multigenerational ADHD. Ultimately, embedding parent-focused supports within BPT may be essential for strengthening treatment impact, durability, and real-world effectiveness for many children and families.

## 1. Introduction

Behavioral parent training (BPT) is a well-established, evidence-based intervention for children with attention-deficit/hyperactivity disorder (ADHD). However, treatment response is variable, with a substantial proportion of children showing attenuated or poorly sustained benefit following intervention [1,2,3]. Parental ADHD symptoms are a critical yet under-addressed factor that may contribute to this variability. ADHD is a highly heritable condition (0.88, [4]) and as many as half of children with ADHD have at least one parent who meets diagnostic criteria for the disorder [5,6]. Growing evidence indicates that parental ADHD is associated with poorer day-to-day implementation of BPT strategies and less robust child treatment response. These adherence challenges likely reflect impairments in parenting processes targeted by BPT (e.g., inconsistent use of contingencies, difficulties with follow-through, harsh or reactive discipline) as well as broader barriers to effective implementation, including executive functioning difficulties, elevated stress, and emotion dysregulation [7,8,9,10]. Importantly, poor parental adherence persists after accounting for child symptom severity and parental depression [8], suggesting that parental ADHD represents a distinct, mechanistically relevant influence on BPT implementation and outcomes.

In the sections that follow, we provide a narrative review of evidence from developmental, clinical, and intervention literatures to propose that psychosocial treatment for childhood ADHD should be conceptualized within an intergenerational framework that considers parental functioning and impairment. We first review research on intergenerational transmission of ADHD-related impairments, parenting behaviors affected by parental ADHD and executive dysfunction, and evidence that parental ADHD moderates engagement with and response to BPT. We next evaluate existing efforts to address parental ADHD-related barriers.

Together, this body of work suggests that improving child outcomes may require interventions that directly target the parental cognitive, regulatory, and organizational processes that interfere with consistent implementation of behavioral parenting strategies. We propose a call to action for the development of next-generation interventions that address parental ADHD in the context of behavioral parent training. While we acknowledge that not all children with ADHD also have parents with symptoms, we contend that parental ADHD is sufficiently common and mechanistically relevant enough that ignoring it systematically limits treatment effectiveness for a meaningful subset of families. Therefore, conceptualizing and treating childhood ADHD within an intergenerational framework may enhance the effectiveness, durability, and real-world impact of psychosocial interventions for many children and families.

## 2. Pathways of Intergenerational ADHD Transmission

Contemporary etiological frameworks of ADHD increasingly conceptualize the disorder as an intergenerational condition, characterized by continuity in symptoms and functional impairment across family members. This perspective is strongly supported by genetic research demonstrating that ADHD is among the most heritable psychiatric conditions, with heritability estimates ranging from 0.75 [11] to 0.88 [4,12]. Nearly half of all children with ADHD have at least one parent who also meets diagnostic criteria [13,14], and parents with ADHD have a greatly increased likelihood of having a child with the disorder. For example, one recent study found that children of parents with ADHD were approximately three times more likely to meet criteria for ADHD themselves relative to children of parents without ADHD [15]. Importantly, however, high heritability does not imply genetic determinism, and contemporary models emphasize that inherited risk is expressed within, and shaped by, the family context through which development unfolds.

Accordingly, rather than conceptualizing symptoms solely within the child, these models emphasize the transmission of ADHD-related vulnerabilities from parents to children through multiple, interacting pathways. A growing body of research indicates that intergenerational patterns of ADHD symptoms and impairment arise from the dynamic interplay between inherited risk and environmental processes, including the parenting environment [16,17]. Within this framework, parenting and other environmental factors are conceptualized as a key mechanism through which genetic risk may be amplified, maintained, or attenuated across development, often dynamically changing over time. Although these perspectives share a common emphasis on heritable risk and parenting processes, intergenerational transmission is understood to occur through multiple distinct pathways, including parenting, genetic, and transactional mechanisms. If the etiology of ADHD is fundamentally intergenerational, psychosocial interventions for childhood ADHD may likewise need to move beyond child-focused approaches to address parental functioning and family-level processes.

Decades of research implicate parenting behaviors in the expression and maintenance of child ADHD symptoms. One comprehensive review found that parents of children with ADHD and related externalizing behaviors, such as oppositional and defiant behaviors, display lower levels of positive parenting (e.g., reduced warmth, praise, and reinforcement), higher levels of inconsistent or lax discipline (e.g., failure to follow through with contingencies, variable rule enforcement), and elevated use of harsh or punitive practices [18]. Longitudinal studies further highlight the negative outcomes of these parenting practices, demonstrating predictive effects of behaviors such as corporal punishment, low parental involvement, and inconsistent discipline on the development and persistence of ADHD and comorbid externalizing symptoms in children [19,20,21,22]. Importantly, these effects extend beyond symptom presence to influence the severity and functional impact of ADHD across development.

Given these associations, parenting behaviors represent a theoretically and empirically supported mechanism through which parental ADHD may influence child symptom development and functional outcomes. Longitudinal research indicates that parental ADHD symptoms prospectively predict higher levels of negative parenting behaviors, including criticism, harsh or inconsistent discipline, and overinvolvement [5,23]. In turn, these parenting behaviors are associated with increases in child ADHD symptoms, externalizing problems, and related impairment across developmental periods spanning early childhood through adolescence [20,22,24]. Cross-sectional research provides converging support for this pathway, demonstrating that parental ADHD symptoms are indirectly associated with greater child ADHD-related impairment via parents’ inconsistent and harsh disciplinary practices [7], supporting a cascading process wherein parental ADHD contributes to maladaptive parenting practices that, in turn, exacerbate child ADHD symptom trajectories.

Rather than operating independently, genetic and environmental influences are closely intertwined in families affected by intergenerational ADHD. One pathway is genetic nurture, whereby parents’ genotypes shape the rearing environment they provide, which in turn influences child symptoms and outcomes. Evidence for genetic nurture in ADHD has been mixed, with some extended twin family and molecular genetic studies reporting small or nonsignificant effects [25,26,27], whereas other recent work suggests a potentially meaningful role for maternal genetic nurture [28].

**Figure 1:**
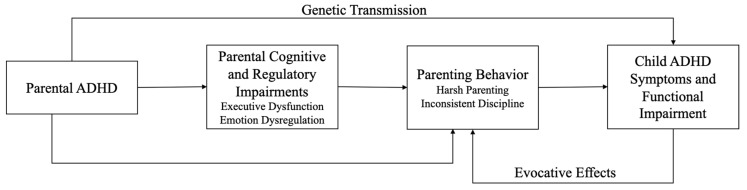
Theoretical model depicting parental ADHD influences on child ADHD symptoms and functional impairment via cognitive and regulatory impairments and their effects on parenting behavior.

A second pathway is evocative genotype–environment correlation, in which children’s genetically influenced ADHD traits elicit specific responses from caregivers. Children’s impulsivity, noncompliance, and emotional reactivity may elicit more reactive, inconsistent, and harsh responses from parents, particularly those with elevated ADHD symptoms [29], intensifying coercive cycles (i.e., reciprocal patterns of escalating interactions in which child misbehavior and parent harsh/inconsistent responses negatively reinforce one another over time [30]). Compounding these dynamics, adults with ADHD are also historically under- and later diagnosed [31] and therefore have fewer years of intervention and supports. As a result, these parents may have had fewer opportunities to develop effective self-regulation and parenting skills, increasing vulnerability to maladaptive parenting patterns. Together, these evocative effects highlight the bidirectional and transactional nature of intergenerational ADHD transmission (see Figure 1).

The association between parental ADHD and suboptimal parenting appears to be driven, at least in part, by underlying deficits in executive functioning [5]. Core executive processes implicated in ADHD (e.g., working memory, inhibitory control, planning and organization, and emotion regulation) are also central to effective parenting [5,32,33,34]. Because consistent and responsive parenting behavior depends on these capacities, weaknesses in executive functioning may undermine parents’ ability to pay close attention to child behavior over time, maintain rules and contingencies in mind, inhibit reactive or emotionally driven responses, and follow through reliably across contexts. Parents with elevated ADHD symptoms may therefore exhibit inconsistency, reduced use of positive reinforcement, and increased reliance on harsh or reactive disciplinary strategies. These executive function challenges likely limit parents’ capacity to provide the structured, predictable environments that are especially important for children with ADHD. In this way, parental ADHD and executive functioning impairments may translate directly into the parenting behaviors that have been shown to exacerbate child ADHD symptoms and impairment.

Finally, cultural and structural contexts also shape the intergenerational pathways of ADHD transmission and influence the operation and expression of genetic, parenting, and transactional processes. Racial, ethnic, linguistic, and socioeconomic disparities in ADHD identification and treatment contribute to systematic underdiagnosis and delayed diagnosis in minoritized populations [35,36], which may postpone availability of supports for both parent and child symptoms. In addition, culturally shaped norms regarding child self-regulation, parenting behavior, and emotional expressiveness may influence how child ADHD-related behaviors are interpreted and responded to within caregiver–child interactions [37,38]. For example, qualitative work with Latino parents of children with ADHD has identified a strong emphasis on *respeto* (i.e., respect [39]), which is associated with higher levels of authoritarian parenting [40]. Taken together, these cultural and structural systems represent cross-cutting influences that shape both the expression of ADHD-related risk and family-level interactions within intergenerational transmission.

*Summary:* Converging evidence from genetic, developmental, and parenting studies supports a cascading, intergenerational model of ADHD in which risk is transmitted through multiple interacting pathways, including parenting behaviors. Within this framework, parental ADHD symptoms and related executive functioning impairments shape the quality and consistency of the home environment, increasing the likelihood of less consistent, more reactive, and less responsive parenting. These behaviors, in turn, potentiate the emergence and persistence of ADHD symptoms and related functional impairment in children. Conceptualizing parenting behavior as a risk mechanism linking parental ADHD to child outcomes highlights a critical and clinically modifiable pathway through which inherited risk is expressed. At the same time, this model situates parenting within a broader network of biological liability and environmental processes, and underscores why parental ADHD may constrain the effectiveness of BPT unless parents’ cognitive and regulatory difficulties are explicitly accommodated or treated.

## 3. Parental ADHD Effects on Behavioral Parent Training

Behavioral parent training (BPT) is one of the most well-established psychosocial interventions for children with ADHD and is widely recommended as a front-line treatment in childhood [41,42,43]. Grounded in social learning and operant theory, BPT aims to reduce ADHD-related symptoms and impairment in children by modifying parent–child interactions and improving parents’ consistent use of positive reinforcement, effective commands, and predictable yet measured consequences. Across decades of randomized controlled trials, BPT has demonstrated robust group-level effects on core ADHD symptoms, externalizing behaviors, social functioning, academic performance, and family functioning [1,41,44]. However, substantial heterogeneity in treatment response remains, with many families showing partial, attenuated, or poorly sustained benefits [1,2].

Because BPT exerts its effects primarily through changes in caregiver behavior, parental characteristics that influence the capacity to sustain behavior change represent theoretically and empirically grounded moderators of treatment response. Research over the past two decades consistently finds that higher levels of parental ADHD symptoms are associated with diminished child improvement following BPT [45,46]. In one of the earliest randomized controlled trials examining the effects of parental ADHD, Sonuga-Barke and colleagues (2002) found that children of mothers with elevated ADHD symptoms exhibited little to no improvement following parent training, whereas children of mothers with low ADHD symptoms demonstrated robust treatment-related improvements. This effect persisted after controlling for maternal depression, parenting self-efficacy, socioeconomic status, and baseline child symptom severity, indicating that parental ADHD symptoms uniquely predict poorer response to BPT rather than reflecting broader psychosocial risk.

Importantly, the association between parental ADHD and poorer child response to BPT appears to reflect attenuated change in the proximal mechanisms (i.e., parenting behaviors and home environment) that BPT is explicitly designed to modify. Parental ADHD symptoms predict less improvement in the parenting behaviors targeted during BPT [47], particularly reductions in negative parenting behaviors and inconsistent discipline [45,48], which subsequently mediate attenuated improvements in child behavior [45]. Similarly, parents with ADHD show reduced effectiveness in modifying the home environment in ways emphasized by BPT. Higher levels of parental ADHD symptoms are associated with greater household disorganization, which in turn mediates relations between parental ADHD and poorer parenting practices [49]. Consistent with this finding, parental ADHD predicts reduced maintenance of treatment gains following the conclusion of structured behavioral interventions, highlighting difficulties sustaining and generalizing behavioral strategies once external supports are withdrawn [50]. These findings suggest that parents with ADHD have particular difficulty establishing and maintaining the structured, predictable, and consistent home environments required for operant-based interventions to function optimally.

Supporting this, Friedman and colleagues (2020) demonstrated that parental ADHD symptoms robustly predict reduced independent use of learned BPT skills in the home environment, even after controlling for child symptom severity, parental anxiety and depression, and socioeconomic factors. An additional study corroborated this pattern, demonstrating that higher parental ADHD symptoms predicted reduced between-session skill use during a BPT program focused on improving social skills for children with ADHD [51]. Notably, parental ADHD is associated with reduced skill use rather than lower session attendance, higher attrition, poorer in-session participation, reduced treatment acceptance, or poorer skill understanding [8,45,51,52], highlighting a specific impairment in the consistent, in vivo implementation of parenting strategies rather than difficulties with general treatment engagement or knowledge acquisition.

Extending these findings, recent qualitative work provides process-level insights into why parents with ADHD struggle to translate BPT knowledge into sustained home implementation. Parents with ADHD and clinicians who regularly deliver BPT similarly described how executive function deficits (e.g., working memory, planning, organization, inhibitory control, emotion regulation, and cognitive flexibility) interfere with the consistent use of parenting strategies in daily life [53]. Parents reported frequent failures to initiate skill use, difficulty remembering and organizing multistep skills (e.g., token economies, and facilitating morning and homework routines), problems inhibiting emotional or habit-driven reactions, and challenges persisting with strategies when child behavior did not improve quickly. Clinicians likewise observed that although parents with ADHD often demonstrate adequate understanding of BPT principles and procedures, they struggle with between-session follow through, maintenance, and generalization of skills, particularly under conditions of stress, child dysregulation, and competing demands. These qualitative findings indicate that the primary barrier to consistent and effective skill implementation among parents with ADHD is not lack of engagement or insight, but rather difficulty executing and sustaining effortful skill use in real-world contexts.

A mechanistically plausible explanation for these findings is that the cognitive and regulatory demands of BPT place substantial load on the very neurocognitive domains most impaired among adults with ADHD, particularly executive functions. Contemporary models conceptualize executive function as encompassing not only ‘cool’ cognitive processes (e.g., working memory, planning, inhibition), but also ‘hot’ regulatory processes such as emotion regulation, motivation, and reward sensitivity, all of which are highly engaged during parenting [32,54,55]. For example, effective implementation of BPT skills requires parents to monitor child behavior, maintain multistep contingencies in mind, inhibit automatic or emotionally driven responses, flexibly adjust strategies, and respond consistently across time and contexts while regulating frustration, coping with child distress, and sustaining motivation in the face of delayed or inconsistent results. Standard BPT approaches rely on parents’ capacity to self-manage these processes, yet the core executive functions frequently impaired in adults with ADHD [56,57] are the same processes required for both cognitive control and emotion regulation during BPT implementation. Beyond moment-to-moment regulation, successful BPT implementation also requires sustained attention, task persistence, and tolerance for delayed or inconsistent reinforcement, as behavioral change in children often unfolds gradually. Adults with ADHD display well-documented difficulties in these areas [58,59,60], which likely further compromise BPT skill use and transfer of learned skills from the therapy setting to the home environment. Emerging theoretical work also suggests that these regulatory processes may be more energetically costly and less sustainable overtime in individuals with ADHD, contributing to fatigue-related declines in executive control [61]. As a result, parents with ADHD may demonstrate intact knowledge yet experience difficulty initiating, organizing, and sustaining skill use outside of structured treatment contexts.

Empirical findings support this mechanistic account. Mazursky-Horowitz and colleagues [62] demonstrated that higher levels of maternal executive functioning, particularly working memory and set shifting, predicted greater observed parental scaffolding during parent–child problem solving tasks. Similarly, parents with high levels of inattentive symptoms displayed poorer observed facilitation, reduced corrective feedback, and less effective social coaching during structured peer interactions [63]. These findings indicate that parental ADHD constrains the real-time executive processes required to provide the structured, contingent, organized, and developmentally appropriate support that many BPT interventions aim to build.

Given that parenting is inherently emotionally salient, difficulties in emotion regulation are particularly likely to present during parent–child interactions. Adults with ADHD experience elevated parenting distress, sleep disruption, and interpersonal strain [64], which are all associated with reduced capacity for patient, consistent, and planful responding. Observational and self-report studies of fathers with childhood ADHD show lower supportive responses to their child’s negative affect and greater reliance on suboptimal emotion socialization practices compared to fathers without ADHD, despite intact warmth and positive intent [65]. Together this literature suggests that parents with ADHD face interacting neurocognitive and affective constraints that interfere with the moment-to-moment organization and persistence required to translate BPT knowledge into consistent behavior change.

*Summary:* Parental ADHD predicts attenuation in children’s treatment gains following BPT, likely due to disruptions in parents’ ability to acquire, consistently implement, and sustain the core parenting strategies taught in BPT. Across randomized trials, mediation analyses, adherence studies, and observational/qualitative research, parental ADHD emerges as a robust predictor of BPT efficacy and is mechanistically informative of impaired skill implementation and sustainment. Executive dysfunction and contextual stressors function as proximal barriers to skill implementation among parents with ADHD, rather than reduced engagement or knowledge. These findings underscore a central implication of an intergenerational framework towards treating childhood ADHD: for a substantial proportion of families, optimizing child outcomes will require next-generation BPT approaches that directly target the parental neurocognitive and regulatory systems required for skill implementation, rather than intervening solely at the level of the child.

## 4. Current Approaches to Addressing Parental ADHD

Given that it is both common for children with ADHD to have a parent with ADHD and for treatment gains to be attenuated in the context of parental ADHD, successful treatment for childhood ADHD must find ways to reduce the interference of parental ADHD symptoms on treatment. A small but growing body of literature has begun to examine whether and how psychosocial interventions for childhood ADHD can be adapted to address the challenges posed by parental ADHD. Collectively, these efforts reflect increasing recognition that parental ADHD is not merely a demographic risk factor for poorer treatment response, but a treatment-relevant characteristic that requires explicit consideration in intervention design.

To date, the most extensively studied strategy for addressing parental ADHD in the context of BPT has been pharmacotherapy. Early work suggested that treating parental ADHD symptoms could, in principle, improve parenting behaviors and thereby enhance child outcomes. In a seminal case study, Evans and colleagues [66] described a mother whose ADHD symptoms appeared to interfere with her ability to benefit from BPT. Using a self-controlled study design, improvements in parenting consistency and child behavior were reported on the days the mother was treated with stimulant medication relative to placebo. This study provided early support for the plausibility of targeting parental ADHD to optimize child-focused interventions.

However, subsequent controlled trials have yielded mixed and often disappointing results. Across studies, stimulant medication reliably reduces parents’ ADHD symptoms, but effects on parenting behaviors are inconsistent. For example, Chronis-Tuscano and colleagues [67,68] found that methylphenidate treatment for parents was associated with improvements in some self-reported parenting behaviors (e.g., inconsistent discipline, corporal punishment) that did not translate to improvements in child behaviors. Similarly, a large randomized trial of atomoxetine (a non-stimulant ADHD medication) found no meaningful improvement in parenting behaviors or child behavior despite parental ADHD symptom reduction [69]. These findings suggest that, although medication may reduce parental ADHD symptoms such as inattention, hyperactivity, and impulsivity, it is unlikely to, on its own, teach or remediate previously underdeveloped executive functions or parenting-relevant skills such as organization and time management which are necessary for effective implementation of BPT skills. Supporting this interpretation, a pilot study comparing maternal stimulant medication to BPT found that mothers receiving medication showed improvement in their own symptoms but not in their parenting skills, while mothers participating in BPT showed greater improvements in parenting skills but limited improvement in their own symptoms [70].

More intensive multimodal approaches using both medication and skills-based treatments have similarly struggled to translate parental symptom improvement into enhanced child outcomes. In a large randomized control trial, Jans and colleagues [71] found that treating maternal ADHD with combined pharmacotherapy (methylphenidate) and manualized DBT-based group therapy prior to BPT did not improve children’s post-BPT externalizing symptoms relative to a control condition, despite improvements in maternal ADHD. Secondary analyses examining mechanisms of change further indicated that the magnitude of maternal ADHD symptom reduction was not associated with improvements in child behavior [72], suggesting that reductions in core parental ADHD symptoms alone are likely insufficient to support improved BPT skill implementation.

Building on these findings, recent work has shifted towards adaptive and sequencing-based approaches to determine whether the timing of parental medication use may improve effects. Pilot data from the first stage of a SMART trial demonstrated that mothers with ADHD are willing to engage in multimodal treatment of their own symptoms as part of a broader strategy for optimizing child outcomes, supporting the feasibility of more complex intervention designs [73]. In a subsequent randomized pilot study examining treatment order, families who received BPT prior to maternal pharmacotherapy showed the greatest improvements in child symptoms, child impairment, and parenting behaviors [74]. However, another study found no added benefit of parental medication when delivered before or after BPT [48]. Thus, the impact of parental pharmacotherapy on parenting and child outcomes remains inconsistent and likely depends on when and how it is integrated with skill-based interventions.

Together, these findings suggest a critical limitation of pharmacological approaches is that reducing core ADHD symptoms does not reliably translate to improved execution of BPT strategies or child outcomes. One likely explanation is that medication improves parents’ general attentional capacity but does not directly target the specific effortful, self-initiated behaviors required for consistent in vivo skill implementation (e.g., planning routines, remembering contingencies, inhibiting reactive responses). Moreover, in many studies, parental medication was administered either prior to [71] or outside of the active BPT phase [48], creating a temporal disconnect that may limit parents’ ability to apply medication-related improvements in cognitive functioning to the real-time demands of parenting interventions.

In contrast to pharmacological approaches, relatively limited research has examined whether directly incorporating parental ADHD-focused skills into BPT can improve outcomes for families with multigenerational ADHD. One of the most rigorous examples comes from Lindström and colleagues [75], who tested a parent training program specifically tailored for adults with ADHD. In this randomized trial, standard BPT was augmented with individualized occupational therapy (OT) designed to support the at-home use of BPT skills. Although the content was individualized, OT sessions commonly focused on identifying barriers to BPT skill use, including organizational challenges, planning routines, and translating session content into daily practice. Parents receiving the adapted intervention showed increased parenting self-efficacy and greater reductions in child externalizing behaviors compared to treatment as usual. Importantly, this approach did not attempt to remediate parental ADHD globally, but instead targeted the functional execution of parenting behaviors, which may explain its comparatively stronger effects relative to pharmacological approaches. Preliminary work integrating other psychosocial strategies into BPT is also promising though limited by small samples and early-stage designs. For example, a small feasibility study found that incorporating Acceptance and Commitment Therapy (ACT) skills for parents into BPT reduced parental stress and increased BPT skill use [76]. While adaptations to behavioral interventions are promising, such approaches have not yet been evaluated in adequately powered trials, nor have they systematically evaluated whether improvements in BPT skill use mediate improved child outcomes.

*Summary:* Emerging research suggests that addressing parental ADHD may represent an important mechanism to improve child outcomes following BPT. Studies examining pharmacological treatment for parents have produced mixed results, with most indicating that medication improves parents’ symptoms without necessarily translating to improved parenting behavior or child outcomes. In contrast, a very limited number of studies provide preliminary evidence for targeted adaptations to BPT programs designed to reduce executive dysfunction or parental stress to support the execution of parental skill use as a possible approach for improving child outcomes.

## 5. Conceptualizing Next-Generation Interventions to Address Parental ADHD

**Figure 2:**
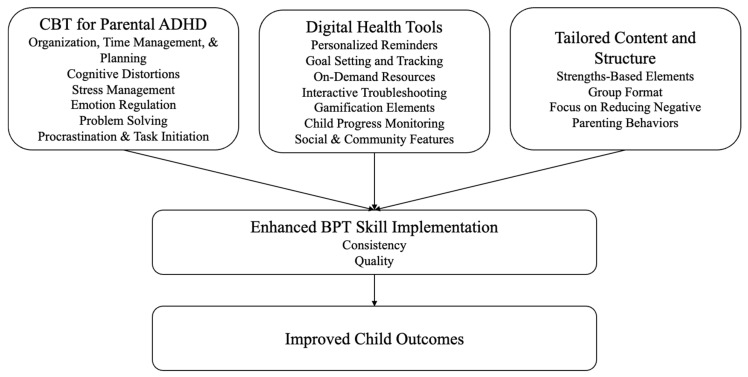
Proposed adaptations to BPT for parents with ADHD.

Across pharmacological and psychosocial approaches to address parental ADHD during BPT interventions, consistent limitations emerge: many interventions target parental ADHD symptoms broadly rather than the specific cognitive and regulatory processes that interfere with BPT skill use. Within these trials, symptom improvement often fails to translate into changes in the consistent, real-world implementation of parenting strategies, which is the proximal mechanism through which BPT exerts its effects. Alternatively, psychosocial approaches show more promise for targeting the moment-to-moment execution of parenting skills by explicitly addressing the proximal barriers to skill use posed by parental ADHD (i.e., executive functioning deficits such as planning, organization, time management, working memory, and emotion regulation difficulties). These observations point to the need for next-generation BPT interventions that integrate targeted, mechanism-focused supports for parents with ADHD directly into the parenting intervention itself (see Figure 2). Such approaches may offer a feasible, scalable, and theoretically coherent pathway towards improving outcomes for families affected by intergenerational ADHD.

### 5.1. Cognitive Behavioral Therapy (CBT) for Parents

One promising approach to improve parenting skill use among families affected by intergenerational ADHD is the incorporation of Cognitive Behavioral Therapy (CBT) strategies into BPT to address the specific cognitive, emotional, and self-regulatory barriers that interfere with parents’ ability to consistently implement learned skills. CBT is an evidence-based approach with demonstrated effectiveness for improving daily functioning among adults with ADHD [77,78,79]. CBT teaches skills such as time management, planning, and emotion regulation, while reducing procrastination and cognitive distortions that can lead to stress, all of which are well-validated barriers that interfere with BPT skill use among parents with ADHD. In large-scale randomized trials, CBT produces reliable benefits in executive functioning, emotion regulation, and daily task execution, particularly in domains such as planning, organization, follow-through, and adaptive coping, which are essential processes for consistent and effective BPT skill implementation [77,79]. Thus, CBT is well positioned to serve as an implementation support for BPT by strengthening the cognitive and regulatory capacities required for BPT skill execution.

Evidence from adjacent literatures further supports the value of integrating CBT strategies into parenting interventions. In families of children with ADHD and elevated parental depression, integrated CBT-BPT interventions have demonstrated improvements in parenting behaviors and child outcomes beyond standard BPT alone. For example, the Integrated Parenting Intervention for ADHD (IPI-A [80]) which combined BPT with CBT for parental depression, improved observed negative parenting, child behavior, and family impairment, despite only modest reductions in parental depressive symptoms. Importantly, evidence suggests that IPI-A exerts its effects by altering the proximal parent cognitive processes rather than primarily reducing parental symptoms. That is, mothers showed reductions in maladaptive cognitions about their children’s behavior (e.g., increased child-crediting attributions), which in turn mediated reductions in observed negative parenting behaviors [81]. This line of work supports the notion that integrating CBT into parenting interventions may enhance child outcomes by modifying the precise barriers to skill use among parents with ADHD, rather than targeting global improvements in parental ADHD symptoms per se. Additional evidence comes from trials of stand-alone CBT interventions for parents of children with ADHD. Wong and colleagues [82] evaluated a group-based CBT program for parents of children with ADHD and found small to moderate improvements in parenting stress, parenting efficacy, maladaptive cognitions, and psychological distress. However, BPT was not provided, and child outcomes were not directly targeted or assessed.

Taken together, the literature suggests that CBT is well-suited to addressing the specific barriers that limit BPT effectiveness among families with intergenerational ADHD. When integrated directly into BPT with the explicit goal of improving BPT skill use (e.g., teaching time management skills to support completion of home practice), CBT strategies can support parents’ ability to translate parenting knowledge into consistent action. These strategies may scaffold planning and organization, enhance task initiation and persistence in the face of frustration, improve regulation of emotional reactivity, and provide techniques to manage both parenting and general life stressors. In this way, CBT functions as an implementation support that strengthens the proximal cognitive and regulatory mechanisms through which BPT operates rather than a standalone treatment for parental ADHD.

### 5.2. Leveraging Digital Health Tools

Digital health tools, such as smartphone applications and other mobile technologies, also offer a promising approach to addressing the noted barriers to BPT implementation experienced by parents with ADHD. Because BPT relies on parents’ consistent, in vivo use of skills, mobile health (mHealth) tools are uniquely positioned to provide temporally relevant supports (e.g., personalized reminders, prompts) at key moments when parenting strategies are intended to be used or when parents may struggle the most. These supports align well with the challenges faced by parents with ADHD during BPT programs, where difficulties often arise not from lack of knowledge but from failures of initiation, consistency, and follow-through. A growing body of literature supports the use of mHealth tools to enhance psychosocial interventions. One meta-analysis [83] found that mHealth technology reliably improves outcomes of psychosocial interventions. Notably, effects were strongest in studies in which mHealth tools supplement rather than replace clinician-delivered interventions. Although some digital parenting programs have focused on improving BPT accessibility by developing asynchronous, online or app-based BPT programs [84,85], such asynchronous programs do not provide supports to address executive and self-regulatory barriers that interfere with consistent BPT skill implementation among parents with ADHD. Overall, adjunctive smartphone tools hold promise for addressing these implementation challenges.

mHealth tools are uniquely positioned to support parents with ADHD because they can provide supports at the point of performance, directly addressing executive functioning and self-regulatory challenges that undermine BPT skill implementation. Automated reminders can reduce memory challenges, and timing can be personalized based on each family’s needs. For example, reminders to deliver rewards for morning routine behaviors can be delivered during weekday mornings, prompting parents to implement reinforcement strategies in real time. In addition, geolocation-based features could provide context-sensitive prompts (e.g., when entering a store or restaurant), supporting parents in implementing behavioral strategies in community settings that are often particularly challenging. On-demand libraries (e.g., parent handouts, brief videos, written guides) can help reduce disorganization by centralizing materials and providing quick access to strategies in moments of need. Gamification elements (e.g., goal tracking, badges) can provide immediate feedback and reinforcement that may be particularly helpful for parents with ADHD given their tendency to exhibit heightened delay discounting [86,87]. Finally, CBT-based troubleshooting supports can help address common barriers as they arise. For example, a troubleshooting wizard on time management can help parents identify competing demands, break parenting tasks into manageable steps, and generate concrete plans for when and how BPT skills will be used. Similarly, brief activities targeting emotion regulation can help parents reframe unhelpful cognitions when frustration or reactivity interferes with skill use. App-based mindfulness interventions for parents of children with ADHD provide promising evidence for this approach. For example, Leitch and colleagues [88] developed brief, app-supported mindfulness practices for parents of children with ADHD to aid in parents’ emotion regulation. While this program did not teach behavioral parenting skills, the high acceptability observed points to promise for mHealth supports when integrated into programs that also address behavioral parenting skills and parents’ executive functioning challenges.

Several digital behavioral parenting tools have been developed and evaluated for childhood ADHD, although most were not designed specifically to address barriers associated with parental ADHD. A systematic review of commercially available ADHD parenting apps concluded that most existing tools emphasize education or symptom monitoring, lack empirical validation, and fail to address the practical, real-world challenges parents face when attempting to consistently implement parenting strategies [89]. Within the broader research landscape, Pfiffner and colleagues [90] developed an adjunctive mHealth tool intended to improve BPT skill use through features such as child behavior tracking, goal monitoring, and an on-demand skills library. While this approach represents an importance advance in leveraging technology to support BPT adherence, the tool was not designed specifically for parents with ADHD and does not include features such as timely, personalized reminders and CBT-based supports to address many of the barriers faced by parents with ADHD.

### 5.3. Augmentations to BPT Content and Structure for Families with Multigenerational ADHD

Although BPT is the front-line non-pharmacological treatment for childhood ADHD, converging research suggests that skill content may require modification for families affected by intergenerational ADHD. Across multiple studies, parental ADHD has shown more robust and consistent associations with negative parenting behaviors (e.g., greater inconsistency, overactivity, harsh discipline) relative to positive parenting behaviors (e.g., praise, warmth [7,20,22,24,29]) Related work further suggests that children’s ADHD-related behaviors preferentially elicit negative, but not positive, parenting responses [21,29,91], consistent with an evocative process whereby child inattention, hyperactivity, and impulsivity are more likely to prompt reactive or harsh responses rather than diminish warmth or support. When parental ADHD symptoms are also present, these child-elicited demands may further amplify parents’ impulsive or emotionally reactive responses, reinforcing coercive parent–child cycles over time that contribute to child externalizing behaviors. Additional evidence implicates negative but not positive parenting behaviors as key mechanisms underlying the poor treatment response observed in families with intergenerational ADHD [45]. In other words, when parents with ADHD experience difficulty decreasing reactive and inconsistent responses, children show minimal behavioral gains during BPT. Together, these findings suggest that, for families with multigenerational ADHD, greater relative emphasis on reducing negative parenting may be necessary to optimize treatment response. This does not negate the importance of positive parenting for parent–child relationship quality and long-term psychosocial well-being; however, for parents with ADHD, reductions in reactive and inconsistent parenting may represent a more proximal and immediately actionable mechanism of change.

The broader parental ADHD literature also points to the value of a strengths-based elements that leverage shared ADHD-related experiences between parents and children. When parents and children both exhibit ADHD symptoms and related traits, parents may possess unique experiential insights into attentional challenges, emotional reactivity, and social frustration that can be harnessed therapeutically. Emerging evidence suggests that parental ADHD does not uniformly undermine parenting intervention engagement or outcomes and may even be associated with enhanced parent–child relationships and connection to treatment. For example, Smit and colleagues [51] found that higher parent ADHD symptoms were associated greater observed collaboration during a structured coaching task, reflecting increased joint-problem solving and child involvement rather than directive parenting. This finding suggests that parents with ADHD may be especially well positioned to empathize with their child’s struggles, validate their experiences, and adopt a collaborative problem-solving approach. In addition, parents with higher levels of ADHD symptoms reported greater perceived social support from other parents while completing a group-based BPT program [51], highlighting the potential of group-based formats. Thus, incorporating opportunities for reflection, peer support, and parent–child collaboration may capitalize on existing strengths within multigenerational ADHD families and enhance engagement, adherence, and child outcomes.

*Summary:* Across pharmacological, psychosocial, and digital approaches, it is increasingly clear that improving parental ADHD symptoms alone is unlikely to sufficiently optimize child outcomes when parents also experience ADHD symptoms. Instead, next-generation BPT interventions for childhood ADHD should be designed to explicitly address the moment-to-moment execution of parenting skills, as ADHD in parents appears to selectively disrupt goal-directed execution of learned BPT skills. For families affected by intergenerational ADHD, effective adaptations will therefore need to integrate mechanism-focused supports for parents within the parenting intervention itself. Such adaptations may incorporate CBT techniques that target parental executive dysfunction and emotion dysregulation that directly impede home practice. These strategies may be supported by leveraging digital health technology, such as smartphone-based apps, that can provide timely, personalized supports at the point of parenting performance to reduce reliance on memory and guide self-regulation in high-demand parenting contexts. In addition, tailored BPT for multigenerational ADHD families should adopt strengths-based elements that leverage shared parent–child experiences and peer support to enhance engagement and persistence. Conceptually, these approaches share a common emphasis on structuring the environment to support parenting skill implementation, aligning with antecedent-based techniques that have demonstrated utility in ADHD populations [92,93]. Together, these strategies offer a coherent, scalable approach to improve adherence, treatment response, and durability of outcomes for families affected by intergenerational ADHD.

## 6. Limitations and Future Directions

Several limitations of the current narrative review and proposed framework warrant consideration. First, the literature on parental ADHD and parenting has been conducted nearly exclusively with mothers, with limited inclusion of fathers or other caregivers who may implement BPT strategies with children (for an exception, see [65]). This constrains generalizability of the findings and recommendations across caregiving contexts. Additionally, much of the existing research relied on relatively homogenous samples, with a predominance of White participants from Western (primarily U.S.) contexts. Because our model conceptualizes parenting as a key mechanism in the intergenerational transmission of ADHD symptoms and treatment-related improvements, it is important to recognize that parenting processes are shaped by broader cultural, contextual, and gender socialization factors. The prominence of mothers and limited representation of diverse racial, ethnic, and cultural groups reduces the ability to generalize how parental ADHD processes operate across diverse family contexts. Future research should prioritize more diverse and representative samples and examine how cultural variation in parenting may influence the role of parental ADHD in BPT implementation and child outcomes.

In addition, this review focuses specifically on BPT, and we do not comprehensively evaluate multicomponent interventions that provide skills to both parent and child. Although such approaches (e.g., Collaborative Life Skills [44], Incredible Years [94]) have demonstrated strong outcomes, it remains unclear whether integrating parent-focused ADHD supports into programs that also provide organizational, emotion regulation, and social skills to children will provide incremental benefit among families with multigenerational ADHD. Future work should directly test whether targeting these parent-level mechanisms enhances implementation and improves child outcomes within both BPT and broader multicomponent interventions. We also provide a narrative review of findings, and our review was not based on a systematic literature search or meta-analytic approach. Conclusions reflect general trends, but future work would benefit from systematic analyses that quantify the strength and consistency of associations between parental ADHD, parenting processes, and child outcomes.

## 7. Conclusions

BPT remains a cornerstone, evidence-based intervention for childhood ADHD, yet its real-world effectiveness is constrained when parents lack the cognitive, regulatory, and organizational capacity to consistently implement learned skills. Parental ADHD is a prevalent and mechanistically important factor shaping both parenting behavior and child treatment response. Specifically, ADHD-related impairments in executive and affective processes undermine the very capacities on which BPT depends, predicting poorer in-home skill use and attenuated child gains. Parental ADHD therefore represents a meaningful, treatment-relevant characteristic rather than merely a demographic risk factor. At the same time, parental ADHD is accompanied by strengths, and parents’ lived experiences with ADHD may confer assets such as empathy, collaborative engagement, and responsiveness to peer support when interventions are designed to leverage these capacities.

Accordingly, we propose an intergenerational rethinking of psychosocial care for childhood ADHD. Much like the familiar airplane instruction to secure one’s own oxygen mask before assisting others, next-generation BPT should routinely assess for parental ADHD and embed targeted implementation supports for parents directly within parenting programs. Promising directions include integrating CBT-informed modules that scaffold planning, organization, and emotion regulation; using digital tools to provide just-in-time reminders and troubleshooting at the point of parenting performance; and tailoring intervention content and emphasis (e.g., prioritizing reduction in reactive/negative parenting) for families affected by multigenerational ADHD. Although pharmacotherapy for parents may serve as a useful adjunct for some families, current evidence suggests that parental ADHD symptom reduction alone is unlikely to be sufficient, and targeted skills-based supports are also necessary to address the cognitive and regulatory challenges associated with parental ADHD.

Together, these insights support a shift toward an explicitly intergenerational model of psychosocial treatment for childhood ADHD where supports for parental cognitive and regulatory processes are directly interwoven into BPT rather than positioned as ancillary components. Although not all families are affected by parental ADHD, its frequency and impact are substantial enough that failing to address it systematically constrains the real-world effectiveness and durability of BPT for many children. Advancing outcomes in childhood ADHD will therefore require next-generation interventions that move beyond child-focused models and more fully account for the dynamic interplay between child behavior and parental functioning.

## Data Availability

No new data were created or analyzed in this study.
